# Simultaneous Determination of 11 Mycotoxins in Maize via Multiple-Impurity Adsorption Combined with Liquid Chromatography–Tandem Mass Spectrometry

**DOI:** 10.3390/foods11223624

**Published:** 2022-11-13

**Authors:** Xin Guan, Yuchao Feng, Decheng Suo, Zhiming Xiao, Shi Wang, Ying Liang, Xia Fan

**Affiliations:** 1 College of Food, Heilongjiang Bayi Agricultural University, Daqing 163000, China; 2 Institute of Agricultural Quality Standards and Testing Technology, Chinese Academy of Agricultural Sciences, Beijing 100081, China

**Keywords:** multiple-impurity adsorption, liquid chromatography–tandem mass spectrometry, mycotoxins, simultaneous detection

## Abstract

In this study, multiple-impurity adsorption purification (MIA) technologies and liquid chromatography–tandem mass spectrometry (LC-MS/MS) were used to establish a method for detecting 11 mycotoxins in maize. The conditions for mass spectrometry and MIA were optimized. Maize was extracted with 70% acetonitrile solution, enriched, and purified using MIA technologies, and then, analyzed via LC-MS/MS. The results showed that the linear correlation coefficients of the 11 mycotoxins were >0.99, the sample recoveries ranged from 77.5% to 98.4%, and the relative standard deviations were <15%. The validated method was applied to investigate actual samples, and the results showed that the main contaminating toxins in maize were aflatoxins (AFs), deoxynivalenol (DON), fumonisins (FBs), ochratoxin A (OTA), and zearalenone (ZEN). Additionally, simultaneous contamination by multiple toxins was common. The maximum detection values of the mycotoxins were 77.65, 1280.18, 200,212.41, 9.67, and 526.37 μg/kg for AFs, DON, FBs, OTA, and ZEN, respectively. The method is simple in pre-treatment, convenient in operation, and suitable for the simultaneous determination of 11 types of mycotoxins in maize.

## 1. Introduction

**Figure 1:**
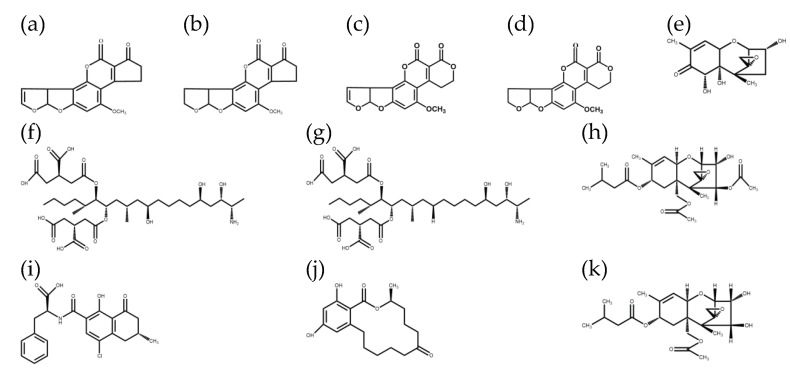
Chemical structures of the 11 mycotoxins: (**a**) aflatoxin B1; (**b**) aflatoxin B2; (**c**) aflatoxin G1; (**d**) aflatoxin G2; (**e**) deoxynivalenol; (**f**) fumonisin B1; (**g**) fumonisin B2; (**h**) T-2 toxin; (**i**) ochratoxin A; (**j**) zearalenone; (**k**) HT-2 toxin.

Maize is one of the most important crops and has rich nutritional value, ranking second in the world in terms of total production. The corn industry is highly associated with farming and animal husbandry. Additionally, maize is an important food source for humans and livestock, as well as a raw material for industry and medicine. With the development of the economy and the improvement of people’s living standards, attention must be paid to research and analysis of the safety of maize products, with mycotoxins having the greatest impact on maize safety. Mycotoxins are toxic metabolites produced by molds in contaminated food, which can seriously affect the health of humans and animals [1]. Maize is one of the most important crops for food and feed. There are many types of mycotoxins in maize and its products, and each mycotoxin has its own harmfulness and uniqueness [2]. Mycotoxins that pose the greatest threat to human health include aflatoxins (AFB1, AFB2, AFG1, and AFG2), deoxynivalenol (DON), fumonisins (FB1 and FB2), T-2 toxin (T-2 and HT-2), ochratoxin A (OTA), and zearalenone (ZEN) (Figure 1) [3,4]; exceeding a certain intake will cause vomiting, diarrhea, organ necrosis, carcinogenicity, teratogenicity, and the induction of immunosuppression and other disorders [5]. The presence of mycotoxins in maize and its products is of global concern. According to the United Nations Food and Agriculture Organization (FAO), 25% of the world’s food is contaminated with mycotoxins [6]. An increasing number of consumers have begun to pay attention to maize safety, and many countries and regions have issued regulations concerning the limit of mycotoxin levels in maize. Hence, the accurate and quantitative detection of mycotoxins in maize has become a research hotspot.

Currently, various mycotoxins are quantified via high-performance liquid chromatography-ultraviolet spectrometry (HPLC-UV) [7,8,9,10] and HPLC–tandem mass spectrometry (LC-MS/MS) [11,12,13,14,15,16,17]. However, HPLC has many disadvantages such as the need for larger sample volumes and longer run times. Even when relying only on LC, it may have low sensitivity and is prone to false positives. LC-MS/MS, with good selectivity and specificity and a low detection limit, has become a popular direction for the study of mycotoxins in recent years, and several methods for detecting multiple mycotoxins in maize have been published [18,19,20,21,22,23,24,25]. The limits of quantification (LOQ) of various toxins are as follows: AFs (0.05~1.6 μg/kg), T-2 (0.05~0.3 μg/kg), DON (0.94~13.6 μg/kg), ZEN (0.5~0.72 μg/kg), OTA (0.03~0.3 μg)/kg), and FBs (1.0∼8.2 μg/kg). Sample cleanup treatment is a crucial part of the analytical assay, which purifies and concentrates the target and can better eliminate substrate interference, improve assay sensitivity, and reduce the detection limit. Currently, sample processing for determining trace mycotoxins by LC-MS/MS primarily uses immunoaffinity methods, solid-phase extraction, and QuEChERS [22,23,24], among other methods. Although the first two methods are effective in removing interfering impurities from samples, they are time-consuming and complex. On the other hand, the QuEChERS method is popular for the detection of pesticide residues but is not very effective for the detection of mycotoxins. In recent years, the technique of purification via multiple-impurity adsorption (MIA) principles has gradually emerged; it mainly involves adsorption of the main interfering impurities in a sample through a variety of functionalized adsorbent materials, effectively removing phospholipids, fats, and some proteins that may be present in the matrix, while leaving the measured substances in the sample solution and achieving purification and enrichment. This has the significant advantages of rapid and simple operation and high detection throughput [25] and has been successfully applied to the simultaneous detection of various drugs in food [26]. Therefore, the development of novel multiple-impurity adsorption methods is necessary to monitor or study multiple mycotoxins in maize.

This study aimed to develop a reliable and simple LC-MS/MS method for detecting 11 mycotoxins in maize, together with the purification of toxins from maize samples using MIA methods. This method was optimized and validated using authentic samples.

## 2. Materials and Methods

### 2.1. Instruments and Reagents

An AB SCIEX QTRAP^®^ 6500+ ultra-HPLC-MS/MS instrument (SCIEX, Redwood City, CA, USA) was equipped with an electrospray ionization source (ESI source). All mycotoxin standard solutions were purchased from Tianjin Alta Technology Co(Tianjin, China). Aflatoxins (aflatoxins B1, B2, G1, and G2) at 100 µg/mL, DON (100 µg/mL), FB1 (100 µg/mL), FB2 (100 µg/mL), HT-2 (100 µg/mL), T-2 (100 µg/mL), OTA (100 µg/mL), and ZEN (100 µg/mL) were used to prepare the combined standards; formic acid (chromatographic purity) was purchased from Sigma (St. Louis, MO, USA), and acetonitrile, methanol (chromatographic purity), and the ChemAlert Guide obtained from Fisher, USA; all other reagents were pure analytical reagents, purchased from Sinopharm Chemical Reagent Co(Beijing, China). The test water was primarily (>18.2 MΩ) prepared via Milli-Q purification in a 0.22 μm nylon membrane (Tianjin Zinteng Experimental Equipment Co., Ltd., Tianjin, China). Maize samples were obtained from several maize-producing areas in Northeast China, Xinjiang, Henan, and Yunnan, and were prepared according to the experimental design.

### 2.2. Purification and Adsorption Material Selection

Thirteen purified adsorbent materials (Table 1) were selected and tested repeatedly to assess the adsorption of 11 mycotoxins in maize species (*n* = 3). After centrifugation of the maize extracts, the supernatant was configured as a 100 ng/mL standard toxin solution. Subsequently, it was purified with 50 mg of adsorbent material, added to 1 mL of the standard toxin solution described above, mixed, vortexed for 5 min, and then, centrifuged for 2 min. The purified supernatant was passed through a 0.22 μm nylon filter membrane and analyzed via LC-MS/MS, while 1 mL of the solution without adsorbent material was used as a blank control. The purification effect was evaluated by comparing the measured values with blank reference value.

### 2.3. Test Method

#### 2.3.1. Standard Solution Preparation

According to the limit requirements and response of the compounds to the instrument, the 11 mycotoxins were divided into a standard stock solution prepared with acetonitrile at a mass concentration of 100 μg/mL. Appropriate amounts of the standard stock solution were pipetted with acetonitrile to prepare a complete mixed standard solution at a mass concentration of 1 μg/mL. Further dilution with acetonitrile was used to prepare an entire mixed classic series of working solutions with mass concentrations of the substances to be measured, consisting of: 0.1, 0.5, 1, 5, 10, 50, 100, and 200 μg/mL.

#### 2.3.2. Formulation of Adsorbent Materials for Multiple-Adsorption Pre-treatment

A total of 10 g BONDESIL-SI(Beijing Puhe Biotechnology Co, Beijing, China), 10 g Esela ^®^ HLB(Hebei Napri Instruments Technology Co。, Shijiazhuan, China) adsorbent materials, and 2 g Cleanert IC-H(Agela, Torrance, CA, USA) adsorbent material were mixed well in a 50 mL conical flask, which was used as the MIA material.

#### 2.3.3. Sample Pre-Treatment

Maize was ground into a powder, passed through a 40-mesh sieve, and placed in a vacuum bag and set aside. We accurately weighed 5 g (to the nearest 0.01 g) of corn sample in a 50 mL centrifuge tube; 20 mL of 70% acetonitrile solution, extracted for 20 min and centrifuged for 5 min; and 1 mL of supernatant to a 10-mL centrifuge tube. A total of 50 mg of MIA material was added for purification, vortexed for 5 min, and then, centrifuged for 2 min. The purified supernatant was filtered through a 0.22 μm nylon membrane and injected into the LC-MS/MS system.

#### 2.3.4. Preparation of Blank Maize Substrate Solution

According to the test method described in Section 2.3.3, all maize samples were tested for toxins, screened for mycotoxin-free maize samples, subjected to extraction experiments, and then, centrifuged to remove the supernatant and set aside.

### 2.4. Method Validation

This selectivity of the method was investigated using 20 mycotoxin-free maize samples. A standard curve was constructed by quantifying the ion chromatographic peak areas against the concentrations of the matrix-spiked standard solutions. The LOQ of each mycotoxin was determined based on the signal-to-noise (S/N) ratio of the quantitative ion chromatographic peak, with S/N = 3 as the limit of detection (LOD) and S/N = 10 as the LOQ, until a sufficient concentration could be measured with acceptable recovery (>70%) and precision (<15%).

Blank maize matrix solutions were used to prepare a variety of mycotoxins in eight standard matrix solutions from low to high concentrations (including the LOQ) at mass concentrations of 0.1, 0.5, 1, 5, 10, 50, 100, and 200 ng/mL, which were determined and analyzed via LC-MS/MS. Linearity studies were performed by analyzing matrix-matched standard solutions (0.5 and 100 ng/mL) in triplicate for 3 days. The calibration curves were based on the selection of eight calibration points (0.1, 0.5, 1, 5, 10, 50, 100, and 200 ng/mL). The lowest and highest concentrations were removed when the correlation coefficient (R) exceeded >0.99. The slope, intercept, and R values were calculated using linear regression.

Authenticity was verified by analyzing the recovery of quality control (QC) samples by precisely aspirating 1 mL of blank maize substrate solution, and then, adding different levels of standard mycotoxin solutions prepared as 2, 5, and 10 ng/mL standards; then, they were assayed as described above with six replicates of each sample for three consecutive days. Precision was expressed via the intra- and inter-RSD obtained in the intra- and inter-day studies. Intra-day analysis was performed six times by measuring QC samples on the same day, while the inter-day study was performed over six days (*n* = 6) by measuring QC, as previously described.

### 2.5. Chromatographic and Mass Spectrometric Conditions

The chromatographic column was a Waters BEH C18 column (100 mm × 3.0 mm, 1.7 µm). The column temperature was 40 °C, the injection volume was 5 µL, and the mobile phase flow rate was 0.4 mL/min. The mobile phase compositions and elution gradients are listed in Table 2**.**

MS was performed using an ESI source (positive and negative ion switching, 1–8 min for the positive ion mode and 7–9 min for the negative ion mode) in multi-reaction monitoring (MRM) mode. The spray voltage was set to 3.2 kV, the ion source temperature was 350 ℃, the gas curtain gas was air, and the collision gas was nitrogen. The flow rate of each gas was adjusted before use so that the MS sensitivity met the detection, segmented acquisition, and acquisition event requirements for a retention time of 1 min; the specific parameters are shown in Table 3.

## 3. Results and Discussion

### 3.1. Optimization of Mobile Phase

**Figure 2:**
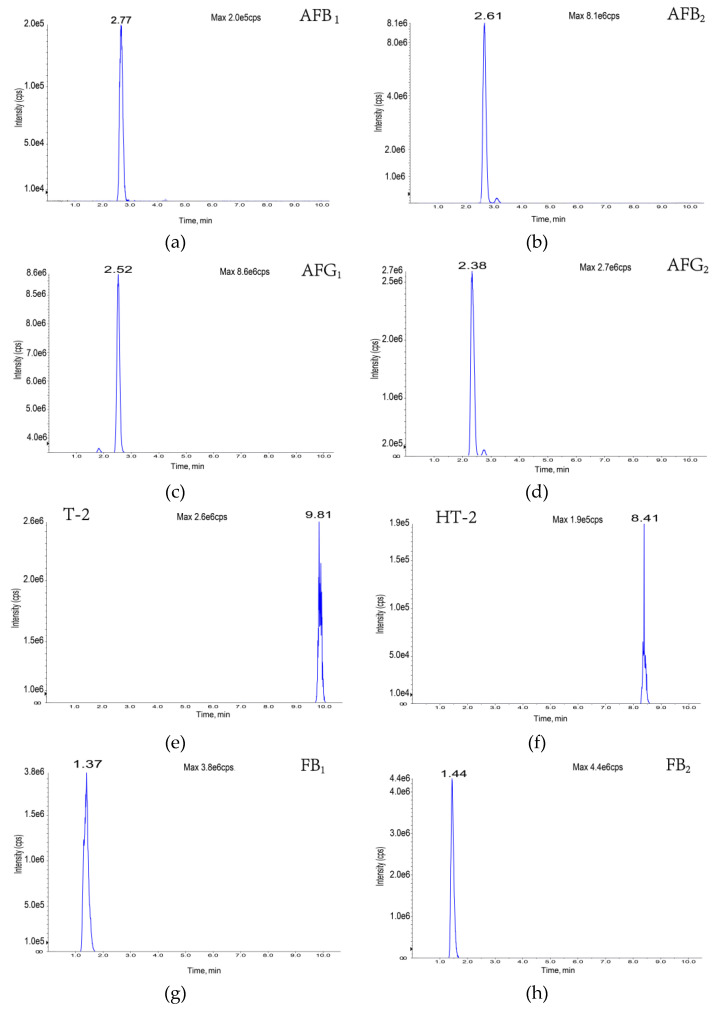

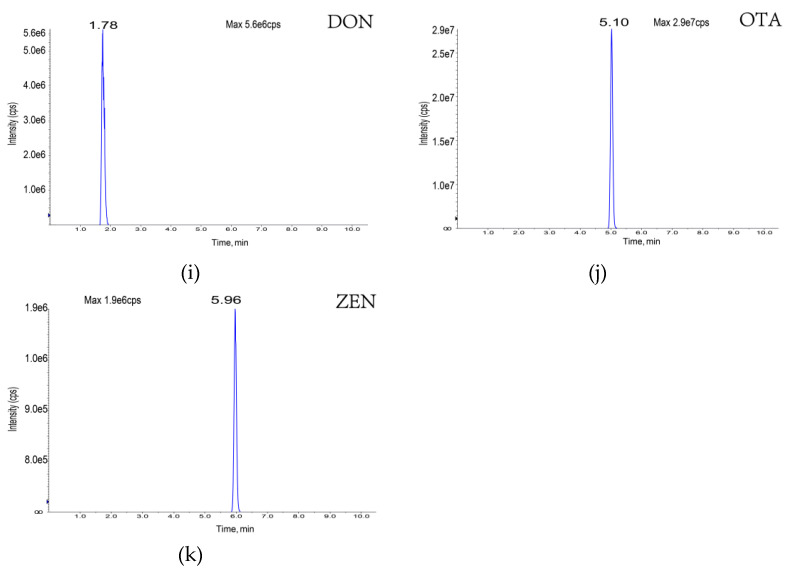
Eleven toxin-specific chromatograms: (**a**) aflatoxin B1; (**b**) aflatoxin B2; (**c**) aflatoxin G1; (**d**) aflatoxin G2; (**e**) T-2 toxin; (**f**) HT-2 toxin; (**g**) fumonisin B1; (**h**) fumonisin B2; (**i**) deoxynivalenol; (**j**) deoxynivalenol; (**k**) zearalenone.

**Figure A1:**
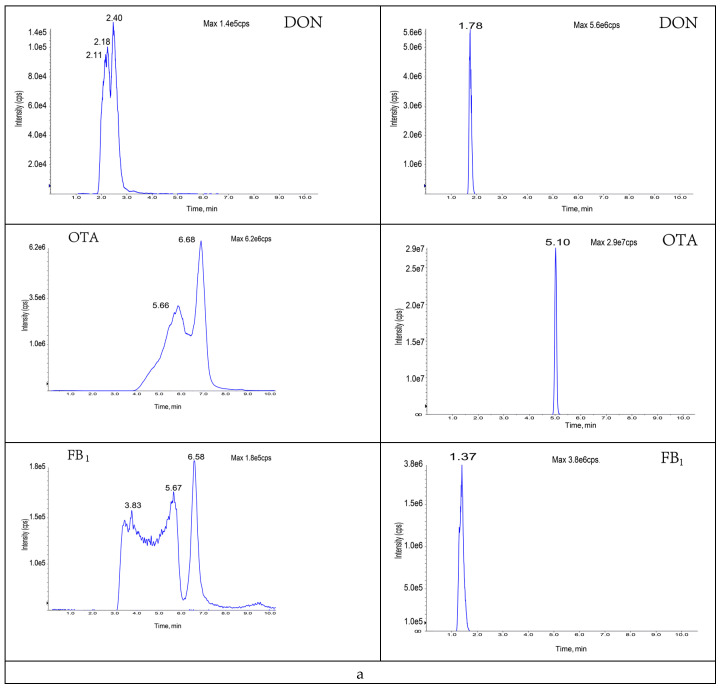

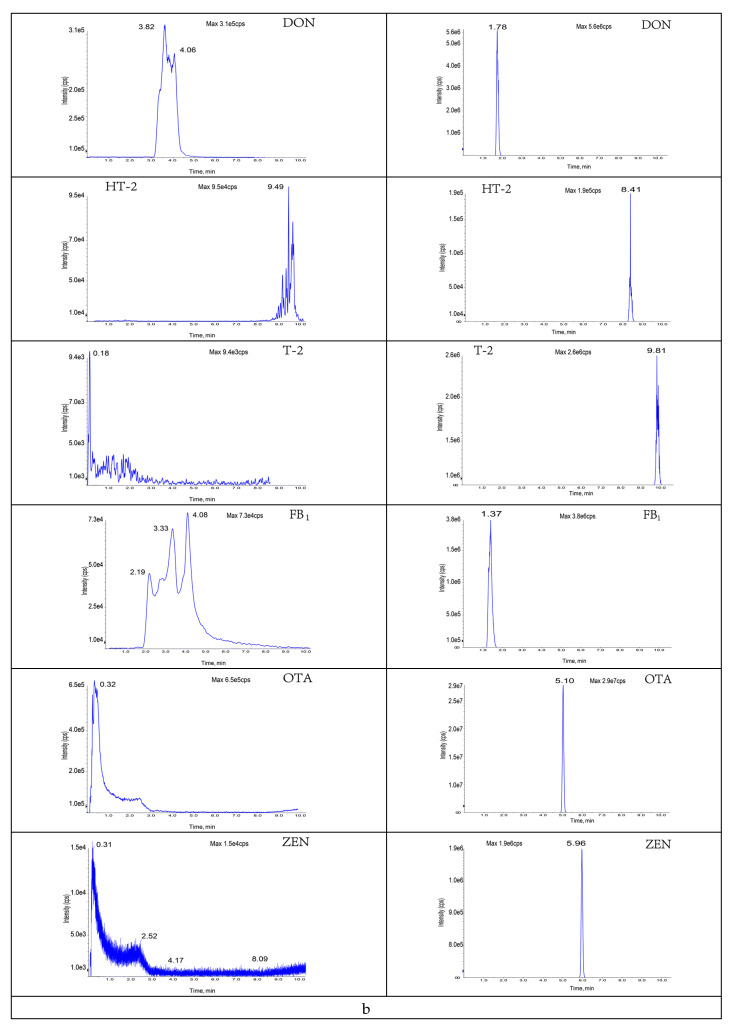

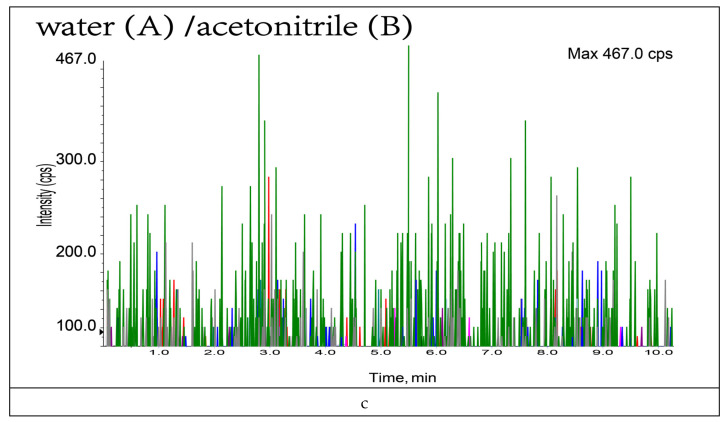
Comparison of different mobile phase toxin chromatograms: (**a**) Comparison between 0.1% formic acid-acetonitrile (B) and acetonitrile (B); (**b**) Comparison between 0.1% formic acid-methanol (B) and acetonitrile (B); (**c**) Comparison between 0.1% formic acid (A) and water (A).

The composition and ratio of the mobile phase not only affect the chromatographic behavior of the target compounds but also influence the ionization efficiency and sensitivity of such targets. In this study, the gradient elution effects of water (A)/acetonitrile (B); 0.1% formic acid (A)/acetonitrile (B); 0.1% formic acid (A)/0.1% formic acid-methanol (B); and 0.1% formic acid (A)/0.1% formic acid-acetonitrile (B) as mobile phases were investigated based on the peak emergence time, separation effect, peak shape, and sensitivity, respectively (Figure A1). The results showed that, under the same gradient elution conditions, 0.1% formic acid solution (A)/acetonitrile (B) was used as the mobile phase at a flow rate of 0.4 mL/min; the peaks were well separated with the highest peak response values at 10 min. The specific mobile phase compositions and elution gradients are listed in Table 2. The specific chromatograms of the 11 mycotoxins are shown in Figure 2.

### 3.2. Optimization of Mass Spectrometry Conditions

High sensitivity was obtained by optimizing the MS parameters of the 11 mycotoxins by injecting standard solutions into an AB SCIEX QTRAP^®^ 6500+ instrument using a continuous microfluidic pump in full scan mode. Among them, AFB1, AFB2, AFG1, AFG2, FB1, FB2, DON, T-2, and OTA showed high response values in the electrospray ionization source (ESI+) mode; hence, the ESI+ method was used for subsequent experiments. In contrast, ZEN showed high response values in ESI- mode; therefore, ESI- mode was used. Two fragment ions with relatively strong signals were selected as qualitative ions for each mycotoxin and the most typical ions were selected as the quantification ions. Further optimization of the parameters, such as the declustering potential (DP) and collision energy (CE), were implemented in the MRM mode of LC-MS/MS (Table 3).

### 3.3. Determination of Sample Pre-Treatment Methods

#### 3.3.1. Optimization and Ratio of the Extraction Solution

Aqueous acetonitrile solutions are often used as extraction solutions to simultaneously detect multiple mycotoxins in existing mycotoxin standards [27,28]. Therefore, we investigated the effects of different acetonitrile-to-water ratios on the extraction of multiple mycotoxins.

According to our experimental results, an 80% acetonitrile solution achieved >60% recovery of AFB1, AFB2, AFG1, AFG2, HT-2, ZEN, and DON. However, only approximately 40–60% recovery was observed for T-2, OTA, and FBs. Increasing the proportion of water in the extraction solution significantly improved the recoveries of T-2, OTA, and FBs. Additionally, the recoveries of T-2, OTA, and FBs in maize reached >60% when 60% acetonitrile was used. However, with an increase in the proportion of water in the extraction solution, the content of the co-extracted impurities in the sample matrix also increased substantially, which increased the difficulty of sample purification and reduced the sensitivity of detection. Therefore, from a practical perspective, a 70% acetonitrile solution was used as the sample extraction solution in this study.

#### 3.3.2. Selection of Purification and Adsorption Materials

**Figure 3:**
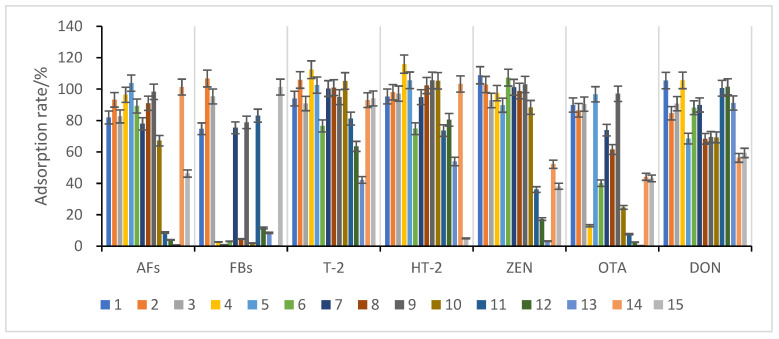
Comparison of the adsorption effect of different purification and adsorption materials on impurities in maize species. Error bars are the standard deviation of the results of 3 measures, like below.

Maize has a complex composition, and matrix effects can significantly impact the accuracy of mycotoxin analysis; therefore, the extracts must be purified. In this study, 13 adsorbent materials (Table 1) were selected for mycotoxin adsorption experiments in maize, and the adsorption results are shown in Figure 3.

The results show that materials 10, 11, 12, and 13 had poor adsorption effects on aflatoxins and ochratoxins. However, they had good enrichment effects on several other toxins. Materials 4, 5, 6, 8, 10, and 14 had poor adsorption effects on fumonisins and ochratoxins but had good adsorption rates for several other toxins. Moreover, material 15 had almost no adsorption effects on HT-2 but had good enrichment effects on other toxins, especially fumonisins. Furthermore, materials 1, 2, 3, 7, and 9 showed significant enrichment for almost every toxin, but only materials 1, 2, and 3 had high adsorption rates for each toxin; materials 1 (BONDESIL-SI), 2 (Cleanert IC-H), and 3 (Esela^®^ HLB) were chosen as enrichment materials for the simultaneous detection of 11 mycotoxins.

Multifunction impurity adsorption (MIA) cleaning is a method based on matrix dispersion solid-phase extraction, which is mainly based on the selection of a variety of functionalized adsorbent materials to adsorb the main interfering impurities in a sample. This effectively removes phospholipids, pigments, and other substances that may be present in the matrix while leaving the measured substances in the sample solution and achieving purification and enrichment, which can save time for sample pre-treatment [25]. According to the adsorption characteristics of different materials such as BONDESIL-SI sorbent, which is based on a bonded reversed-phase (with high-purity irregular silica gel as the matrix and end-group capping treatment), a typical reversed-phase extraction retention mechanism has excellent strength retention properties for non-polar compounds and can remove lipids from samples. Cleanert IC-H is based on the principle of reversed-phase adsorption and ion exchange and can effectively remove impurities such as organic matter and ionic impurities from the sample, avoiding contamination of the ion chromatography column by pollutants and their effect on separation. Esela^®^ HLB packing is prepared via a unique co-polymerization technique containing a specific ratio of hydrophilic and hydrophobic groups; the hydrophobic divinylbenzene structure retains non-polar compounds, and the hydrophilic N-vinylpyrrolidone system retains polar compounds. A filler was used to reduce the effects of pigments and other impurities (e.g., metal ions) in the samples. The mixture could effectively remove interference from various impurities in maize and achieve better purification results. Therefore, BONDESIL-SI, Cleanert IC-H, and Esela^®^ HLB were selected as the multiple-mechanism impurity adsorption materials for the next steps of the study.

#### 3.3.3. Study of Proportion of Treatment Materials Used

**Figure 4:**
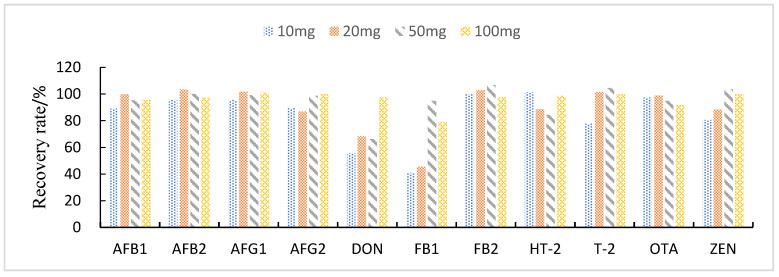
Effect of different BONDESIL-SI additions on the recovery of 11 fungal toxins.

**Figure 5:**
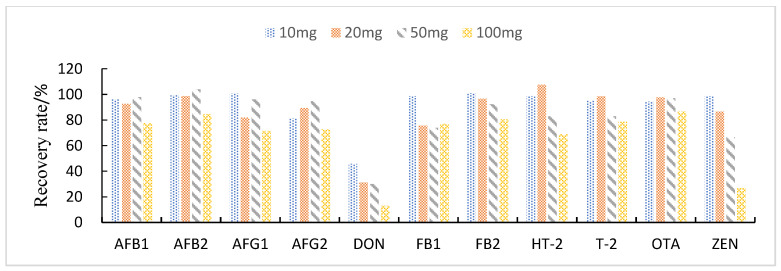
Effect of different Esela^®^ HLB additions on the recovery of 11 fungal toxins.

**Figure 6:**
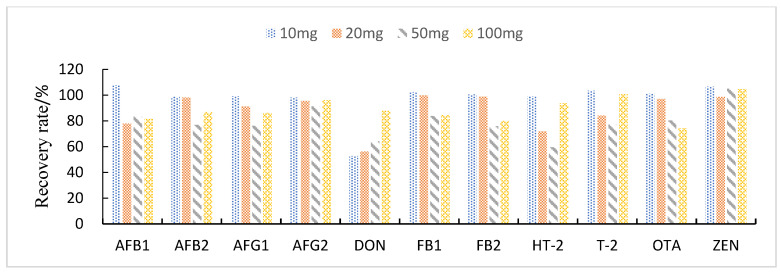
Effect of different Cleanert IC-H additions on the recovery of 11 fungal toxins.

This study compared the effects of different solid adsorbent additions (10, 20, 50, and 100 mg) on the purification of impurities in 1 mL of maize extract. The results showed that when BONDESIL-SI and Esela^®^ HLB were added at 50 mg, most of the mycotoxins achieved high recoveries. Additionally, increasing the amount of sorbent did not significantly improve the purification effect and recovery of sample impurities (Figure 4 and Figure 5). Cleanert IC-H could effectively remove pigments and other impurities from the extracted solution. However, it adsorbed certain target compounds as its addition increased, leading to poor recovery (Figure 6). Therefore, the amount of adsorbent material to be added was set to 50 mg. When the adsorbent adsorption purification was added, it effectively removed the influence of interfering substances in the matrix on the chromatographic peaks of the analytes to be measured. Simultaneously, it reduced damage from impurities to the instrument and column.

**Figure 7:**
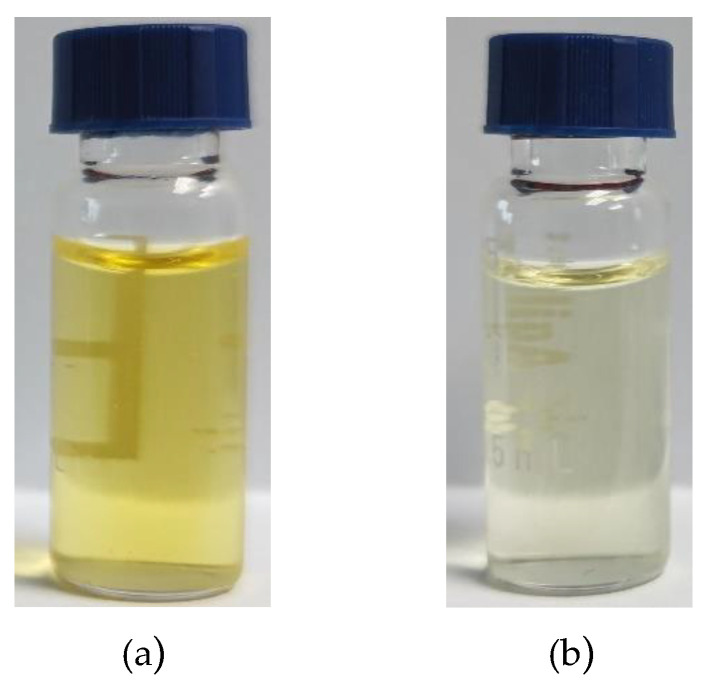
Comparison of maize extract before and after purification: (**a**) Before purification; (**b**) After purification.

From the matrix purification effects of the above experiments, the ratio of BONDESIL-SI, Esela^®^ HLB, and Cleanert IC-H was 5:5:1, and the purification effect is shown in Figure 7. Almost all 11 of these mycotoxins exhibited good purification effects, meaning they could effectively adsorb impurities and reduce the matrix effect.

### 3.4. Validation of the Proposed Method

To evaluate the effectiveness of the method described here, its quantitative characteristics were considered under optimal conditions, including the LOD (S/N = 3), LOQ (S/N = 10), and precision (expressed as the relative standard deviation). The results are presented in Table 4 and Table 5.

In this study, the ratio of the slope of the matrix standard curve to that of the solvent standard curve was used to evaluate the matrix effect (matrix effect = blank matrix standard response value/pure solvent average response value × 100%. When the matrix effect was more significant than 1, it showed a matrix-enhancement effect; when the matrix effect was <1, it was a matrix-inhibition effect). The results showed that AFs, FBs, OTA, and ZEN had strong matrix-enhancement effects, whereas DON, T-2, and HT-2 had strong matrix-inhibition effects. The parameters of the method were investigated using matrix-matching curves to improve assay accuracy and precision.

Calibration curves were plotted using matrix-matched standard solutions by adding eight concentrations (each concentration was repeated thrice) to the maize blank. The matrix-matched calibration curves of the analytes showed good linearity with a correlation coefficient of ≥0.9905. The LOD and LOQ values were 0.02–0.8 μg/kg and 0.06–2.5 μg/kg, respectively. The LOD and LOQ values were lower than those of the previously mentioned methods because of the better selectivity and sensitivity of LC-MS/MS.

The recoveries and intra- and inter-day reproducibility in the maize matrices are summarized. The recoveries in maize ranged from 77.5% to 98.4%, with an intra-day relative standard deviation (RSDs) of 0.84% to 11.9% and inter-day RSDs of 1.84% to 13.2%; the standard deviations were ≤15% for the resulting analytes. This indicates that the recoveries of the LC-MS/MS conducted in this test were not significantly different from those of previously reported methods and met the recovery requirements for mycotoxin detection in maize at a certain level, demonstrating the ability of the technique to measure analytes in the presence of possible interference in the samples.

The methodological properties of this test method were compared with those reported in the literature (Table 6). It was found that each of the many methods has advantages, and that these disadvantages should not be ignored. The new purification methods established in these experiments, using the principle of multiple-impurity adsorption, simplify the purification process to a certain extent and shorten purification time. The method is simple, accurate, and has a high sensitivity, which is advantageous for practical applications.

### 3.5. Determination of the Proposed Method on Maize

In this step, 100 maize samples purchased from different producers were analyzed using the developed method to determine the concentrations of the studied mycotoxins. Among the 100 maize samples, mycotoxins were detected in 30 batches; the main contaminating toxins in maize were AFB1, DON, FB1, OTA, and ZEN, and simultaneous contamination with multiple toxins was more common (Table 7). The maximum concentrations were 77.65 μg/kg for AFB1, 1294.19 μg/kg for DON, 20,0212.41 μg/kg for FB1, 9.67 μg/kg for OTA, and 526.37 μg/kg for ZEN.

## 4. Conclusions

A method was developed to purify maize samples using multiple-impurity adsorption combined with LC-MS/MS to simultaneously determine the abundance of 11 mycotoxins. The process meets the analytical requirements for mycotoxins in maize, and the sample extracts are directly filtered and cleaned for machine detection, which is a simple and rapid pre-treatment operation that can be used for the daily testing and monitoring of relevant samples. Traditional pre-treatment and purification methods require multiple steps. In contrast, this method requires only “one-step” filtration of the sample extract to complete the sample purification process, significantly reducing the sample pre-treatment time and improving the detection efficiency. By optimizing the ratio of the extract to the mobile phase, the sensitivity and accuracy of the method were guaranteed. This provides a simple, rapid, accurate, and reliable method for the simultaneous determination of 11 mycotoxins in maize.

## Figures and Tables

**Table 1:** Name of purification and adsorption materials.

Number	Name	Material Type	Specification
1	BONDESIL-SI	Silica gel	400 μm
2	Cleanert IC-H	Ion-exchange resin	40–60 μm
3	Esela^®^ HLB	Hydrophilic–Lipophilic Balance	40–60 μm
4	Aluminum oxide	Alkaline alumina	100–200 μm
5	C18	Silica gel-bonded octadecyl	50 μm
6	PSA	Silica gel-bonded N-propylethylenediamine	40–60 μm
7	SCX	Sodium sulfonate bonded on silica gel	50 μm
8	BONDESIL-FL	Flori silica	200 μm
9	SAX	Silica gel-bonded halogenated quaternary Ammonium Salt	40 μm
10	SLE	Diatomite	80–100 mesh
11	Pesti Carb	Activated carbon	120–400 mesh
12	CMCNs	Carboxylated multiwalled carbon nanotubes	8–15 nm
13	MCNs	Multiwalled carbon nanotubes	<8 nm
14	Z-Sep+	Silica matrix surface double-bonded with C18 and Z-Sep	100–200 μm
15	CarbonX	Carbon fiber	120–400 mesh

**Table 2:** Chromatographic conditions for detection of 11 mycotoxins by AB SCIEX QTRAP^®^ 6500+ LC-MS/MS.

Time	Flow	Acetonitrile (B) %	0.1% Formic Acid (A) %
0.0	0.4	20	80
0.5	0.4	20	80
3.0	0.4	40	60
6.0	0.4	95	5
7.0	0.4	95	5
11.0	0.4	95	5
11.1	0.4	20	80

**Table 3:** Mass spectrometry conditions for LC-MS/MS detection of 11 mycotoxins.

Toxin	Molecular Formula	Molecular Weight	Precursor Ion (M/Z)	Retention Time (min)	Cone Voltage (V)	Collision Energy (EV)	Fragment Ion (M/Z)
Aflatoxin B1	C_17_H_12_O_6_	312	313	2.77	160	35	241, 285
Aflatoxin B2	C_17_H_14_O_6_	314	315	2.61	160	35	259, 287
Aflatoxin G1	C_17_H_12_O_7_	328	329	2.52	150	35	243, 200
Aflatoxin G2	C_17_H_14_O_7_	347	331	2.38	160	35	189, 245
Deoxynivalenol	C_15_H_20_O_6_	296	296	1.78	60	17	249, 203
Fumonisin B1	C_34_H_59_NO_15_	721	722	1.37	80	49	352, 334
Fumonisin B2	C_34_H_59_NO_14_	705	706	1.44	80	49	337, 355
T-2 toxin	C_24_H_34_O_9_	466	484	9.81	40	25	185, 305
HT-2 toxin	C_22_H_32_O_8_	424	442	8.34	40	16	263, 245
Zearalenone	C_18_H_22_O_5_	318	317	5.96	60	-35	131, 175
Ochratoxin A	C_20_H_18_CINO_6_	408	404	5.10	−140	35	239, 193

**Table 4:** Linear equations and regression coefficients of the matrix-matched standard calibration curves (*n* = 3).

Feed Matrix	Correlation Coefficient (R^2^)	Slope (b)	Intercept (a)	LOD (μg/kg)	LOQ (μg/kg)
Aflatoxin B1	0.9996	2306 ± 108	−1669 ± 92	0.02	0.06
Aflatoxin B2	0.9995	27,976 ± 811	−409 ± 38	0.04	0.12
Aflatoxin G1	0.9993	14,258 ± 971	1030 ± 42	0.05	0.15
Aflatoxin G2	0.9991	5767 ± 563	601 ± 12	0.05	0.15
Deoxynivalenol	0.9992	409 ± 38	80 ± 8	0.02	0.06
Fumonisin B1	0.9952	1468 ± 142	−1843 ± 136	0.15	0.5
Fumonisin B2	0.9905	2798 ± 146	−5651 ± 178	0.2	0.6
T-2 toxin	0.9978	1761 ± 33	−1770 ± 95	0.2	0.6
HT-2 toxin	0.9948	216 ± 8	−173 ± 4	0.8	2.5
Zearalenone	0.998	15,344 ± 169	−12,071 ± 947	0.08	0.3
Ochratoxin A	0.9991	104,722 ± 7013	−106,855 ± 4179	0.02	0.06

**Table 5:** Recovery and precision of different mycotoxins spiked in maize (*n* = 6).

Toxin	2 μg/kg			5 μg/kg			10 μg/kg		
	Recovery (%)	Inter-RSD (%)	Intra-RSD (%)	Recovery (%)	Inter-RSD (%)	Intra- RSD (%)	Recovery (%)	Inter RSD (%)	Intra-RSD (%)
Aflatoxin B1	89.8	10.8	8.9	87.2	8.3	8.8	92.5	5.3	8.6
Aflatoxin B2	77.5	3.5	7.8	79.2	3.8	3.7	80.9	2.3	3.5
Aflatoxin G1	86.7	4.6	8.2	80.4	4.3	4.7	90.4	9.1	8.5
Aflatoxin G2	83.9	7.7	9.6	83.9	7.7	7.0	87.8	3.7	4.5
Deoxynivalenol	87.9	8.4	8.2	87.1	9.0	12.3	89.1	4.3	8.9
Fumonisin B1	85.2	6.6	8.6	84.4	5.9	8.6	85.8	4.5	8.9
Fumonisin B2	84.1	11.9	13.2	88.3	11.0	10.2	89.5	6.8	5.2
T-2 toxin	91.0	9.3	10.6	88.8	10.5	10.3	93.7	5.7	7.3
HT-2 toxin	91.4	5.9	6.0	90.5	5.4	7.1	92.8	9.8	10.8
Zearalenone	92.3	6.0	6.8	93.0	0.8	1.8	98.4	4.0	3.7
Ochratoxin A	93.6	5.4	6.4	94.2	3.8	3.8	97.9	3.6	4.4

**Table 6:** Comparison of methodological properties of different detection methods.

Method	Mycotoxin Type	Advantages	Disadvantages	LOQ	Reference
Methods based on QuEChERS	10	Quick, easy, cheap, effective, rugged, safe	Rely on high-precision instruments; mainly used for the analysis of pesticides; cumbersome preparation time.	0.38~25 μg/kg	[26]
	8			1.0~200 μg/kg	[29]
Methods based on multi-antibody immunoaffinity	12	High specificity and selectivity, safe	Longer cleaning times and lower specificity; need for special process equipment; high cost of immunoaffinity columns; complex procedures involving degreasing or separate ESI+ and ESI- monitoring.	0.3~118.7 μg/kg	[22]
	6			0.1~50 μg/kg	[30]
	5			0.1~1.0 μg/kg	[31]
SPE	9	Simple and inexpensive	Cumbersome and time-consuming to operate.	0.03~2.12 μg/kg	[19]
	9			0.3~195.7 μg/kg	[32]
Multiple-impurity adsorption	11	Quick, easy, cheap, strong, effective, safe	/	0.06~2.5 μg/kg	Methods in this paper

/ None.

**Table 7:** Mycotoxin contamination of analyzed maize samples (*n* = 100).

Toxin	Lowest (μg/kg)	Highest (μg/kg)	Allowable Limit *	Exceeding the Standard Samples (%)
Aflatoxin B1	0.17	77.65	20	7
Aflatoxin B2	0.11	5.00	20	0
Aflatoxin G1	0.03	0.16	20	0
Aflatoxin G2	0.08	0.59	20	0
Deoxynivalenol	9.17	1294.19	1000	4
Fumonisin B1	92.28	200,212.41	/	/
Fumonisin B2	37.45	89,834.45	/	/
T-2 toxin	0.23	3.52	/	/
HT-2 toxin	3.92	17.38	/	/
Zearalenone	0.17	526.37	60	22
Ochratoxin A	0.02	9.67	5.0	1

* Toxin limit standards based on GB 2761-2017; / None.

## Data Availability

Data sharing is not applicable to this article.

## References

[1] Lattanzio V.M.T., Della Gatta S., Suman M., Visconti A. (2011). Development and in-house validation of a robust and sensitive solid-phase extraction liquid chromatography/tandem mass spectrometry method for the quantitative determination of aflatoxins B1, B2, G1, G2, ochratoxin A, deoxynivalenol, zearalenone, T-2 and HT-2 toxins in cereal-based foods. Rapid Commun. Mass Spectrom..

[2] Miklós G., Angeli C., Ambrus Á., Nagy A., Kardos V., Zentai A., Kerekes K., Farkas Z., Jóźwiak Á., Bartók T. (2020). Detection of Aflatoxins in Different Matrices and Food-Chain Positions. Front. Microbiol..

[3] Abdallah M.F., De Boevre M., Landschoot S., De Saeger S., Haesaert G., Audenaert K. (2018). Fungal Endophytes Control *Fusarium graminearum* and Reduce Trichothecenes and Zearalenone in Maize. Toxins.

[4] Majer-Baranyi K., Adányi N., Székács A. (2021). Biosensors for Deoxynivalenol and Zearalenone Determination in Feed Quality Control. Toxins.

[5] Hajnal E.J., Kos J., Malachová A., Steiner D., Stranska M., Krska R., Sulyok M. (2020). Mycotoxins in maize harvested in Serbia in the period 2012–2015. Part 2: Non-regulated mycotoxins and other fungal metabolites. Food Chem..

[6] Van der Fels-Klerx H.J.I., Adamse P., Punt A., Van Asselt E.D. (2018). Data Analyses and Modelling for Risk Based Monitoring of Mycotoxins in Animal Feed. Toxins.

[7] Eskola M., Kos G., Elliott C.T., Hajslova J., Mayar S., Krska R. (2020). Worldwide contamination of food-crops with mycotoxins: Validity of the widely cited “FAO estimate” of 25. Crit. Rev. Food Sci. Nutr..

[8] Algammal A.M., Elsayed M.E., Hashem H.R., Ramadan H., Sheraba N.S., El-Diasty E.M., Abbas S.M., Hetta H.F. (2021). Molecular and HPLC-based approaches for detection of aflatoxin B1 and ochratoxin A released from toxigenic Aspergillus species in processed meat. BMC Microbiol..

[9] Zhang Y., Pei F., Fang Y., Li P., Zhao Y., Shen F., Zou Y., Hu Q. (2019). Comparison of concentration and health risks of 9 Fusarium mycotoxins in commercial whole wheat flour and refined wheat flour by multi-IAC-HPLC. Food Chem..

[10] Murali H.S. (2021). Development and evaluation of multiplex PCR for detection of T-2 and zearalenone producing *Fusarium* spp.. Lett. Appl. Microbiol..

[11] Zareshahrabadi Z., Karimirad M., Pakshir K., Bahmyari R., Motamedi M., Nouraei H., Zomorodian K. (2021). Survey of aflatoxins and ochratoxin A contamination in spices by HPLC-based method in Shiraz, Southern of Iran. Environ. Sci. Pollut. Res..

[12] Peng H., Chang Y., Baker R.C., Zhang G. (2020). Interference of mycotoxin binders with ELISA, HPLC and LC-MS/MS analysis of aflatoxins in maize and maize gluten. Food Addit. Contam. Part A.

[13] Dhanshetty M., Thorat P., Banerjee K. (2021). High-Throughput Analysis of Aflatoxins in Cereals, Nuts, and Processed Products Involving Automated Immunoaffinity Cleanup and Inline HPLC–Fluorescence Detection. J. AOAC Int..

[14] Nakhjavan B., Ahmed N.S., Khosravifard M. (2020). Development of an Improved Method of Sample Extraction and Quantitation of Multi-Mycotoxin in Feed by LC-MS/MS. Toxins.

[15] Meyer H., Skhosana Z.D., Motlanthe M., Louw W., Rohwer E. (2019). Long Term Monitoring (2014–2018) of Multi-Mycotoxins in South African Commercial Maize and Wheat with a Locally Developed and Validated LC-MS/MS Method. Toxins.

[16] Malachová A., Stránská M., Václavíková M., Elliott C.T., Black C., Meneely J., Hajšlová J., Ezekiel C.N., Schuhmacher R., Krska R. (2018). Advanced LC–MS-based methods to study the co-occurrence and metabolization of multiple mycotoxins in cereals and cereal-based food. Anal. Bioanal. Chem..

[17] Di Marco Pisciottano I., Imperato C., Urbani V., Guadagnuolo G., Imbimbo S., De Crescenzo M., Soprano V., Esposito M., Gallo P. (2020). T-2 and HT-2 toxins in feed and food from Southern Italy, determined by LC-MS/MS after immunoaffinity clean-up. Food Addit. Contam. Part B.

[18] Kudumija N., Vulić A., Lešić T., Vahčić N., Pleadin J. (2020). Aflatoxins and ochratoxin A in dry-fermented sausages in Croatia, by LC-MS/MS. Food Addit. Contam. Part B.

[19] Hickert S., Gerding J., Ncube E., Hübner F., Flett B., Cramer B., Humpf H.-U. (2015). A new approach using micro HPLC-MS/MS for multi-mycotoxin analysis in maize samples. Mycotoxin Res..

[20] Wang Y., Xiao C., Guo J., Yuan Y., Wang J., Liu L., Yue T. (2013). Development and Application of a Method for the Analysis of 9 Mycotoxins in Maize by HPLC-MS/MS. J. Food Sci..

[21] Park J., Kim D.-H., Moon J.-Y., An J.-A., Kim Y.-W., Chung S.-H., Lee C. (2018). Distribution Analysis of Twelve Mycotoxins in Corn and Corn-Derived Products by LC-MS/MS to Evaluate the Carry-Over Ratio during Wet-Milling. Toxins.

[22] Kappenberg A., Juraschek L. (2021). Development of a LC–MS/MS Method for the Simultaneous Determination of the Mycotoxins Deoxynivalenol (DON) and Zearalenone (ZEA) in Soil Matrix. Toxins.

[23] Solfrizzo M., Gambacorta L., Bibi R., Ciriaci M., Paoloni A., Pecorelli I. (2018). Multimycotoxin Analysis by LC-MS/MS in Cereal Food and Feed: Comparison of Different Approaches for Extraction, Purification, and Calibration. J. AOAC Int..

[24] Woo S.Y., Ryu S.Y., Tian F., Lee S.Y., Park S.B., Chun H.S. (2019). Simultaneous Determination of Twenty Mycotoxins in the Korean Soybean Paste Doenjang by LC-MS/MS with Immunoaffinity Cleanup. Toxins.

[25] Seo H., Jang S., Jo H., Kim H., Lee S., Yun H., Jeong M., Moon J., Na T., Cho H. (2021). Optimization of the QuEChERS-Based Analytical Method for Investigation of 11 Mycotoxin Residues in Feed Ingredients and Compound Feeds. Toxins.

[26] Cheng S., Wei Z., Zhiming X., Yang L., Xia F. (2021). Trace analysis and identification of 33 sulfonamides and sulfonamide potentiators in eggs by ultrahigh-performance liquid chromatography coupled with quadrupole-high-field orbitrap high-resolution mass spectrometry. Anal. Methods.

[27] Mateus A.R.S., Barros S., Pena A., Silva A.S. (2021). Development and Validation of QuEChERS Followed by UHPLC-ToF-MS Method for Determination of Multi-Mycotoxins in Pistachio Nuts. Molecules.

[28] (2011). Appendix B. Determination of Aflatoxins, Zearalenone and T-2 in Feeds-Liquid Chromatography-Tandem Mass Spectrometry.

[29] (2017). Appendix C. National Food Safety Standards Limits for Mycotoxins in Food.

[30] Shephard G.S., Berthiller F., Burdaspal P., Crews C., Jonker M., Krska R., Macdonald S., Malone R., Maragos C., Sabino M. (2012). Developments in mycotoxin analysis: An update for 2010–2011. World Mycotoxin J..

[31] Annunziata L., Stramenga A., Visciano P., Schirone M., De Colli L., Colagrande M.N., Campana G., Scortichini G. (2017). Simultaneous determination of aflatoxins, T-2 and HT-2 toxins, and fumonisins in cereal-derived products by QuEChERS extraction coupled with LC-MS/MS. Anal. Bioanal. Chem..

[32] Iram W., Anjum T., Abbas M., Khan A.M. (2014). Aflatoxins and ochratoxin A in maize of Punjab, Pakistan. Food Addit. Contam. Part B.

